# Association between gamma-glutamyl transferase and diabetes factors among elderly nonobese individuals

**DOI:** 10.1097/MD.0000000000041913

**Published:** 2025-03-21

**Authors:** Chih-Tsueng He, Fang-Yu Chen, Chun-Heng Kuo, Chung-Yu Lin, Dee Pei, Pietro Pitrone, Jin-Shuen Chen, Chung-Ze Wu

**Affiliations:** a Department of Internal Medicine, Division of Endocrinology and Metabolism, School of Medicine, College of Medicine, Taipei Medical University, Taipei City, Taiwan, R.O.C.; b Department of Internal Medicine, Division of Endocrinology and Metabolism, Shuang Ho Hospital, Taipei Medical University, New Taipei City, Taiwan, R.O.C.; c Department of Internal Medicine, Division of Endocrinology and Metabolism, Fu Jen Catholic University Hospital, School of Medicine, College of Medicine, Fu Jen Catholic University, New Taipei City, Taiwan, R.O.C.; d Department of Cardiology, Graduate Institute of Business Administration, Fu Jen Catholic University Hospital, Fu Jen Catholic University, New Taipei City, Taiwan, R.O.C.; e Radiology Department, Papardo Hospital, Messina, Italy; f Kaohsiung Veterans General Hospital, Kaohsiung City, Taiwan, R.O.C.; g Institute of Precision Medicine, National Sun Yat-sen University, Kaohsiung City, Taiwan, R.O.C.; h Department of Medicine, Division of Nephrology, Tri-Service General Hospital, National Defense Medical Center, Taipei City, Taiwan, R.O.C.

**Keywords:** first-phase insulin secretion, gamma-glutamyl transferase, glucose effectiveness, insulin resistance, second-phase insulin secretion

## Abstract

Type 2 diabetes mellitus is a significant health concern among elderly individuals in Taiwan, and liver dysfunction, particularly nonalcoholic fatty liver disease, is prevalent in this population. Gamma-glutamyl transferase (γ-GT), a key enzyme involved in glutathione metabolism, has been linked to metabolic disorders, including insulin resistance (IR) and diabetes. However, its association with insulin secretion phases (first-phase insulin secretion, FPIS; second-phase insulin secretion, SPIS) and glucose effectiveness (GE) remains unclear. This study aimed to investigate these relationships in elderly nonobese Chinese individuals. A total of 10,933 participants (5082 men and 5851 women) aged ≥ 65 years were enrolled. Participants were classified based on metabolic syndrome (MetS) status and γ-GT quartiles. Blood samples were analyzed for γ-GT, fasting plasma glucose, insulin resistance, and lipid profiles. The study used established equations to calculate IR, FPIS, SPIS, and GE. Pearson correlation analysis and statistical models were applied to assess the associations. 768 men and 794 women had MetS. Those with MetS had higher IR, FPIS, SPIS, γ-GT levels, and lower GE. Higher γ-GT levels were significantly associated with MetS components and increased IR, FPIS, SPIS, and decreased GE. GE had the strongest negative correlation (*r* = -0.198 for men, −0.158 for women), followed by positive correlations with IR (*r* = 0.183 for men, 0.132 for women), SPIS (*r* = 0.099 for men, 0.060 for women), and FPIS (*r* = 0.028 for men, 0.048 for women). γ-GT was positively associated with IR, FPIS, and SPIS but negatively correlated with GE in elderly individuals. Among the 4 diabetes factors, GE exhibited the strongest correlation with γ-GT, followed by IR, SPIS, and FPIS. These findings highlight the need for further research into the role of γ-GT in glucose metabolism and potential clinical implications for elderly nonobese Chinese populations.

## 1. Introduction

Due to the national health insurance policy, Taiwan has witnessed an increase in the average life expectancy. Officially, Taiwan has been classified as an aging society since 2014, with 11.7% of the population over 65 years old. The prevention and management of age-related disorders, including type 2 diabetes mellitus (T2DM), cardiovascular diseases, and stroke, have become important concerns for healthcare providers and governments.^[1]^

Nonalcoholic fatty liver disease (NAFLD) is a clinicopathological term that encompasses a range of conditions from simple triglyceride accumulation in hepatocytes (hepatic steatosis) to hepatic steatosis with inflammation (steatohepatitis), fibrosis, and cirrhosis.^[2]^ NAFLD, along with T2DM, constitutes a significant public health concern. In addition to its function in nutrition metabolism, the liver plays a critical role in glucose homeostasis through gluconeogenesis, insulin clearance, and insulin resistance. Gamma-glutamyl transferase (γ-GT) is an enzyme that is especially high in concentrations in the liver. It plays a crucial role in the metabolism of glutathione, an antioxidant that helps protect cells from damage. Clinically, elevated γ-GT levels serve as a marker of liver dysfunction.^[3]^ It is worth noting that NAFLD is closely associated with elevated serum γ-GT levels, obesity, insulin resistance (IR), and hyperinsulinemia.^[4,5]^ Notably, in patients with T2DM, serum γ-GT levels have been found to be positively related to visceral adipose tissue and hepatic fat content but not to subcutaneous adipose tissue.^[6]^ These associations could be explained by the relationship between γ-GT and oxidative stress as well as hepatic steatosis.^[7–9]^

Impaired insulin secretion and increased IR are widely considered the underlying pathophysiology of T2DM. However, 2 other important factors have often been overlooked. There are 2 phases of insulin secretion: first-phase insulin secretion (FPIS) and second-phase insulin secretion (SPIS). Second, glucose effectiveness (GE) refers to metabolic physiology describing the ability of glucose itself, independent of insulin, to regulate blood glucose levels. Specifically, GE reflects the capacity of glucose to be taken up by cells or to suppress hepatic glucose output without relying on insulin. Low GE indicates a reduced ability to regulate blood sugar, an increased demand for insulin production, and a higher risk of developing T2DM. Studies have shown that in normal subjects, both insulin-mediated and non-insulin-mediated glucose uptake accounts for approximately 50% when plasma glucose is 11.1 mmol/L.^[10]^ These 4 factors, namely impaired insulin secretion, increased IR, FPIS, and GE, can be collectively referred to as diabetes factors (DF).

Despite previous studies demonstrating the tight association between γ-GT and IR, reduced β-cell function, and deterioration of glucose tolerance,^[11,12]^ little is known about the relationship between γ-GT and other DFs, especially in the elderly population. This knowledge gap may be attributed to difficulties in measuring these factors. Therefore, the present study aimed to address 2 key questions: (1) the relationships between γ-GT and DFs, and (2) which of the DFs is most strongly associated with γ-GT. To answer these questions, we enrolled elderly participants.

## 2. Materials and methods

### 2.1. Study subjects

Participants were randomly enrolled from the MJ Health Screening Center, the Outpatient Department of Cardinal Tien Hospital, and Tri-Service General Hospital in Taiwan. Inclusion criteria required all participants to be able to clearly express themselves and to be adults aged 65 years or older. The study period spanned from January 1999 to December 2018. The study protocol was approved by the institutional review board of each institution (Approval No: TSGH-100-05-246 and CTH-100-2-5-036), adhering to the provisions of the Declaration of Helsinki. All study participants remained anonymous and informed consent was obtained from each participant. Individuals who were obese (body mass index [BMI] ≥ 25 kg/m^2^) or taking medications known to affect blood pressure, glucose, and lipid levels were excluded. Participants who have a habit of alcoholic drinking or known liver disease, except for NAFLD, were also excluded from the study. Participants were categorized into those with metabolic syndrome (MetS) and those without MetS based on the criteria of the World Health Organization.^[13]^ Finally, a total of 5082 men and 5851 women were enrolled in the study. Among the men, there were 768 patients with MetS (MetS(+)) and 4314 without MetS (MetS(-)). There were 794 women with MetS(+) and 5057 without MetS(-).

On the day of the study, senior nursing staff obtained the participants’ medical history, including information on current medications. Thorough questionnaires and complete physical examinations were conducted. Waist circumference (WC) was measured horizontally at the natural waist level, identified as the level at the hollow molding of the trunk when it was laterally concave. BMI was calculated by dividing the subject’s body weight (kg) by the square of their height (m). The systolic blood pressure (SBP) and diastolic blood pressure (DBP) were measured by nursing staff using standard mercury sphygmomanometers on the right arm of each subject while seated. After a 10-hour fast, blood samples were drawn from the antecubital vein for biochemical analysis. Plasma was separated from the blood within 1 hour and stored at 30°C for the analysis of fasting plasma glucose (FPG) and lipid profiles. FPG was measured using the glucose oxidase method (YSI 203 glucose analyzer; Yellow Springs Instruments, Yellow Springs, USA). Total cholesterol and triglyceride levels were measured using a dry, multilayer analytical slide method with a Fuji Dri-Chem 3000 analyzer (Fuji Photo Film, Tokyo, Japan). Serum high-density lipoprotein cholesterol (HDL-C) concentration was analyzed using an enzymatic cholesterol assay following dextran sulfate precipitation. Serum γ-GT was performed using a CX7 biochemistry analyzer (Beckman, Fullerton, CA).

The equations for calculating IR, FPIS, SPIS, and GE were as follows: It is important to note that all the units are international units. The numbers 1 and 2 represent men and women, respectively. The publication information for each equation is given in parentheses.

The equations used to calculate IR, FPIS, SPIS, and GE are as follows: A brief report is provided to assess the reliability of these equations. Approximately 70% of the sample participants were used to construct the equations, while the remaining 30% were used for external validation, ensuring accountability of the equations.

1. IR: A total of 327 subjects were enrolled in this equation, which estimates insulin resistance using an insulin suppression test. The correlation (*r*) between the obtained and calculated GE was 0.581 (*P* < .001).^[14]^

IR = log(1.439 + 0.018 × sex − 0.003 × age + 0.029 × BMI − 0.001 × SBP + 0.006 × DBP + 0.049 × TG − 0.046 × HDL-C − 0.0116 × FPG) × 10^3.333^

2. FPIS: A total of 186 subjects were included in this equation, which measures first-phase insulin secretion by using an intravenous glucose tolerance test with frequent sampling. The correlation value (*r*) between the measured and calculated GE values was 0.671 (*P* < .000).^[15]^

FPIS = 10^(1.477–0.119 × FPG + 0.079 × BMI −^^ 0.523 × HDL-C)^

3. SPIS: A total of 82 participants were included in this equation, which measures second-phase insulin secretion through a modified glucose infusion test with a low dose. The correlation value (*r*) between the measured and calculated GE was 0.65 (*P* = .002).^[16]^

SPIS = 10^(−2.4–0.088 × FPG + 0.072 × BMI)^

4. GE: A total of 227 participants were included in this equation, which measures glucose effectiveness using a constant sampled intravenous glucose tolerance test. The correlation value (*r*) between the measured and calculated GE was 0.43 (*P* = .001).^[17]^

GE = (29.196–0.103 × age − 2.722 × TG − 0.592 × FPG) × 10^−3^

### 2.2. Statistical analysis

The data are presented as mean ± standard deviation. Participants were grouped according to the presence of MetS and γ-GT quartiles. We categorized subjects into quartiles based on their γ-GT levels, arranged from the lowest to the highest values. To normalize the distribution, γ-GT levels were transformed logarithmically. This transformation allowed us to define groups as Log γ-GT1 through Log γ-GT4, from the lowest quartile to the highest quartile. By applying this method, we aimed to enhance the statistical validity of comparisons and better interpret the variations in γ-GT levels among the subjects. Student *t* test was used to assess differences in continuous data between MetS(+) and MetS(−) groups. One-way analysis of variance was used to evaluate differences in demographic data, clinical parameters, and DFs with FPG in the γ-GT quartiles. The Bonferroni test was used for the post hoc analysis. Pearson correlation analysis was used to examine the correlation between γ-GT levels and DFs. A general linear model was used to determine the differences between the 4 slopes and FPG. All statistical tests were 2-sided, and a *P*-value of <.05 was considered statistically significant. Statistical analysis was performed using SPSS 10.0 for Windows (SPSS, Chicago, IL). The significance of the differences between the 2 slopes was evaluated using Chris’ calculator.

## 3. Results

### 3.1. Clinical characteristics of participants in MetS(−) and MetS(+)

The clinical characteristics and 4 DFs of the MetS(−) and MetS(+) groups are presented in Table 1. Both sexes in the MetS(+) group exhibited higher age, BMI, WC, SBP, DBP, FPG, triglyceride, cholesterol, γ-GT, FPIS, SPIS, IR, and lower HDL-C and GE levels.

**Table 1:** Demographic data of the participants without (MetS(-)) and with (MetS(+)) metabolic syndrome.

	Men			Women		
	MetS (－)	MetS (+)	*P*	MetS (－)	MetS (+)	*P*
n	4341	768		5057	794	
γ-GT (U/L)	25.7 ± 31.6	30.5 ± 28.7	<.001	19.6 ± 24.8	24.5 ± 38.8	.001
Log γ-GT	1.305 ± 0.258	1.382 ± 0.269	<.001	1.193 ± 0.250	1.269 ± 0.271	<.001
Age (yr)	65.9 ± 5.7	66.7 ± 6.0	<.001	64.3 ± 4.8	67.1 ± 6.1	<.001
BMI (kg/m^2^)	22.5 ± 1.3	23.3 ± 1.2	<.001	22.5 ± 1.4	23.2 ± 1.2	<.001
Waist circumference (cm)	80.9 ± 5.4	85.4 ± 5.9	<.001	74.0 ± 4.9	79.5 ± 5.8	<.001
SBP (mm Hg)	127.7 ± 19.2	141.7 ± 17.0	<.001	129.3 ± 19.9	144.4 ± 18.0	<.001
DBP (mm Hg)	74.8 ± 11.0	82.1 ± 11.1	<.001	73.2 ± 11.3	79.4 ± 11.0	<.001
FPG (mg/dL)	93.3 ± 5.1	95.0 ± 5.3	<.001	93.0 ± 5.0	94.2 ± 5.1	<.001
Triglyceride (mg/dL)	102.5 ± 47.0	187.7 ± 71.7	<.001	107.5 ± 49.0	187.4 ± 67.4	<.001
HDL-C (mg/dL)	53.2 ± 13.8	41.0 ± 8.1	<.001	62.9 ± 14.9	46.3 ± 10.0	<.001
FPIS (μU/min)	94.6 ± 46.5	143.7 ± 46.8	<.001	70.7 ± 36.1	123.8 ± 42.0	<.001
SPIS (pmol/mmol)	0.060 ± 0.014	0.066 ± 0.013	<.001	0.060 ± 0.014	0.066 ± 0.013	<.001
IR (10^-4^/min/pmol/L)	3.669 ± 0.017	3.690 ± 0.017	<.001	3.669 ± 0.017	3.688 ± 0.016	<.001
GE (10^-2^/dL/min/kg)	0.016 ± 0.002	0.013 ± 0.002	<.001	0.016 ± 0.002	0.013 ± 0.002	<.001

Data are shown as the mean ± standard deviation. MetS = metabolic syndrome, BMI = body mass index, WC = waist circumference, SBP = systolic blood pressure, DPB = diastolic blood pressure, FPG = fasting plasma glucose, HDL-C = high-density lipoprotein cholesterol, γ-GT = γ-glutamyl transpeptidase, Log γ-GT = log transformation of γ-glutamyl transpeptidase, FPIS = first-phase insulin secretion, SPIS = second-phase insulin secretion, IR = insulin resistance, GE = glucose effectiveness.

### 3.2. Components of MetS according to quartiles of γ-GT

γ-GT quartiles were used to determine the components of MetS. Similar trends to those shown in Table 1 were observed among the 4 groups divided by quartiles of Log γ-GT (Log γ-GT 1–4), indicating a significant association. Higher FPIS, SPIS, IR, age, BMI, WC, SBP, DBP, FPG, triglyceride, and lower HDL-C and GE levels were consistently linked to increasing levels of γ-GT (Table 2).

**Table 2:** The anthropometric variables of subjects in different log transformed γ-glutamyl transpeptidase groups.

	Log γ-GT1		Log γ-GT2		Log γ-GT3		Log γ-GT4		Total	*P*
Men										
n	1273		1273		1273		1272		5091	
γ-GT (U/L)	11.16 ± 1.87	**,***,****	16.26 ± 1.50	*,***,****	22.63 ± 2.58	*,**,****	55.74 ± 51.68	*,**,***	26.44 ± 31.18	<.001
Log γ-GT	1.04 ± 0.08	**,***,****	1.21 ± 0.04	*,***,****	1.35 ± 0.05	*,**,****	1.67 ± 0.22	*,**,***	1.32 ± 0.26	<.001
Age (yr)	67.03 ± 6.09	**,***,****	66.09 ± 5.74	*,****	65.55 ± 5.45	*	65.32 ± 5.39	*,**,***	66.00 ± 5.71	<.001
BMI (kg/m^2^)	22.38 ± 1.36	**,***,****	22.62 ± 1.33	*,***,****	22.78 ± 1.35	*,**	22.82 ± 1.33	*,**	22.65 ± 1.35	<.001
WC (cm)	80.44 ± 5.66	**,***,****	81.34 ± 5.67	*,***,****	82.00 ± 5.53	*,**,****	82.68 ± 5.71	*,**,***	81.61 ± 5.7	<.001
SBP (mm Hg)	128.89 ± 19.68	****	129.28 ± 19.77		129.77 ± 18.52		131.30 ± 20.09	*	129.81 ± 19.54	.011
DBP (mm Hg)	74.85 ± 11.24	****	75.79 ± 11.45	****	76.01 ± 11.11		76.97 ± 11.37	*,**	75.91 ± 11.31	<.001
FPG (mg/dL)	93.38 ± 5.10		93.56 ± 5.08		93.44 ± 5.31		93.71 ± 5.20		93.52 ± 5.17	.397
Triglyceride (mg/dL)	93.80 ± 45.45	**,***,****	110.01 ± 53.97	*,***,****	121.60 ± 59.94	*,**,****	136.14 ± 69.33	*,**,***	115.38 ± 59.86	<.001
Log triglyceride	1.93 ± 0.18	**,***,****	2.00 ± 0.19	*,***,****	2.04 ± 0.20	*,**,****	2.08 ± 0.21	*,**,***	2.01 ± 0.20	<.001
HDL-C (mg/dL)	51.39 ± 13.21		51.31 ± 13.30		50.58 ± 13.40	****	52.11 ± 15.27	***	51.35 ± 13.83	.051
FPIS (μU/min)	97.29 ± 48.39	***,****	101.01 ± 49.68	***	106.54 ± 50.05	*,**	103.21 ± 50.53	*	102.01 ± 49.77	<.001
SPIS (pmol/mmol)	0.058 ± 0.014	**,***,****	0.061 ± 0.013	*,***,****	0.062 ± 0.010	*,**	0.062 ± 0.013	*,**	0.061 ± 0.014	<.001
IR (10^-4^/min/pmol/L)	3.666 ± 0.018	**,***,****	3.671 ± 0.017	*,***,****	3.674 ± 0.020	*,**,****	3.676 ± 0.019	*,**,***	3.672 ± 0.018	<.001
GE (10^-2^ dL/min/kg)	0.0163 ± 0.0016	**,***,****	0.0159 ± 0.0018	*,***,****	0.0156 ± 0.0001	*,**,****	0.0152 ± 0.0023	*,**,***	0.0158 ± 0.0020	<.001
Women										
n	1457		1456		1456		1456		5825	
γ-GT (U/L)	8.83 ± 1.60	**,***,****	12.63 ± 1.11	*,***,****	17.30 ± 1.79	*,**,****	42.50 ± 47.59	*,**,***	20.31 ± 27.21	<.001
Log γ-GT	0.94 ± 0.09	**,***,****	1.10 ± 0.04	*,***,****	1.24 ± 0.04	*,**,****	1.54 ± 0.23	*,**,***	1.20 ± 0.25	<.001
Age (year)	64.69 ± 5.10		64.66 ± 5.12		64.73 ± 5.03		64.55 ± 5.06		64.66 ± 5.08	.803
BMI (kg/m^2^)	22.41 ± 1.36	**,***,****	22.65 ± 1.36	*	22.71 ± 1.35	*	22.75 ± 1.37	*	22.63 ± 1.36	<.001
WC (cm)	74.10 ± 5.45	***,****	74.57 ± 5.24	***,****	75.14 ± 5.41	*,**	75.30 ± 5.40	*,**	74.78 ± 5.40	<.001
SBP (mm Hg)	129.73 ± 20.21	***,****	131.17 ± 20.80		132.39 ± 20.40	*	132.29 ± 19.62	*	131.40 ± 20.28	.001
DBP (mm Hg)	72.95 ± 10.98	***,****	73.93 ± 11.74		74.69 ± 11.43	*	74.69 ± 11.42	*	74.07 ± 11.42	<.001
FPG (mg/dL)	92.59 ± 5.22	**,***,****	93.11 ± 4.87	*	93.32 ± 5.08	*	93.53 ± 4.85	*	93.14 ± 5.02	<.001
Triglyceride (mg/dL)	103.08 ± 49.47	**,***,****	114.22 ± 54.06	*,***,****	123.21 ± 59.91	*,**,****	132.86 ± 65.83	*,**,***	118.34 ± 58.67	<.001
Log triglyceride	1.97 ± 0.18	**,***,****	2.02 ± 0.19	*,***,****	2.05 ± 0.20	*,**,****	2.08 ± 0.20	*,**,***	2.03 ± 0.20	<.001
HDL-C (mg/dL)	61.53 ± 15.31	***	60.96 ± 15.58		59.85 ± 14.99	*	60.30 ± 15.90		60.66 ± 15.46	.019
FPIS (μU/min)	73.56 ± 40.42	**,***,****	77.65 ± 41.05	*	80.11 ± 40.54	*	80.34 ± 42.46	*	77.91 ± 41.21	<.001
SPIS (pmol/mmol)	0.059 ± 0.014	**,***,****	0.061 ± 0.014	*	0.062 ± 0.014	*	0.062 ± 0.014	*	0.061 ± 0.014	<.001
IR (10^-4^/min/pmol/L)	3.668 ± 0.017	**,***,****	3.671 ± 0.018	*,***,****	3.674 ± 0.017	*,**	3.675 ± 0.018	*,**	3.672 ± 0.018	<.001
GE (10^-2^ dL/min/kg)	0.0163 ± 0.0017	**,***,****	0.0160 ± 0.0018	*,***,****	0.0157 ± 0.0020	*,**,****	0.0154 ± 0.0022	*,**,***	0.0158 ± 0.0020	<.001

Data are shown as mean ± standard deviation. BMI = body mass index, WC = waist circumference, SBP = systolic blood pressure, DPB = diastolic blood pressure, FPG = fasting plasma glucose, HDL-C = high-density lipoprotein cholesterol, γ-GT = γ-glutamyl transpeptidase, Log γ-GT = log transformation of γ-glutamyl transpeptidase, FPIS = first-phase insulin secretion, SPIS = second-phase insulin secretion, IR = insulin resistance, GE = glucose effectiveness.

* *P* < .05 against Log γ-GT1.

** *P* < .05 against Log γ-GT2.

*** *P* < .05 against Log γ-GT3.

*** *P < .05 against Log γ-GT4.

### 3.3. Relationship between γ-GT and 4 DFs

**Figure 1.**
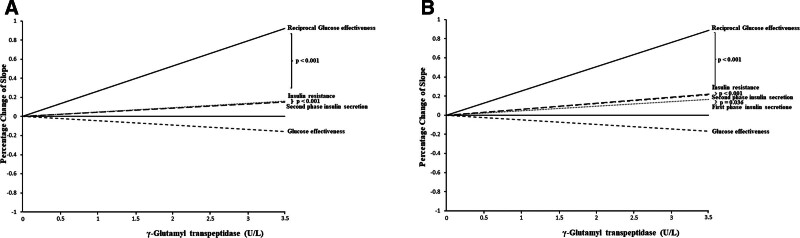
Comparison of the different insulin parameters in (A) men and (B) women according to the increased log transformed γ glutamyl transpeptidase (γ-GT).

Table 3 shows the results of the simple correlations between γ-GT and the 4 DFs. It is worth noting that GE had a negative relationship with γ-GT, whereas the other 3 factors showed a positive relationship. Additionally, GE exhibited the highest correlation coefficient (*r* = −0.198 for men and −0.158 for women, *P* < .001), indicating the strongest association with γ-GT. IR (*r* = 0.183 for men and 0.132 for women, *P* < .001) and SPIS (*r* = 0.099 for men and 0.060 for women, *P* < .001) followed in strength, whereas FPIS (*r* = 0.028, *P* = .045 for men and *r* = 0.048, *P* < .001) demonstrated the weakest correlation. Figure 1 provides a graphical representation of these relationships. In men, there was a significant difference in the slope between IR and FPIS but not between GE, IR, GE, and SPIS. Among women, significant differences existed between GE, IR, and both FPIS and SPIS, while no significant difference was observed between FPIS and SPIS.

**Table 3:** Simple correlation between γ-glutamyl transpeptidase and 4 different insulin parameters.

	Men		Women	
	*r*	*P*	*r*	*P*
FPIS	0.028	.045	0.048	<.001
SPIS	0.099	<.001	0.060	<.001
IR	0.183	<.001	0.132	<.001
GE	－0.198	<.001	－0.158	<.001

FPIS = first-phase insulin secretion, GE = glucose effectiveness, IR = insulin resistance, SPIS = second-phase insulin secretion.

## 4. Discussion

As stated in the Introduction, both T2DM and NAFLD are prevalent in Taiwan. Additionally, γ-GT is associated with glucose dysregulation. Therefore, this study aimed to explore the relationship between γ-GT, IR, FPIS, SPIS, and GE in elderly individuals. The findings of our study indicate that elderly, nonobese Chinese subjects with higher levels of γ-GT exhibited higher IR, FPIS, SPIS, and lower GE.

The association between γ-GT and T2DM incidence is well-known.^[18]^ Furthermore, a strong correlation between γ-GT and MetS has been confirmed.^[6]^ Therefore, the positive correlation between γ-GT and IR observed in our study was not surprising. Similar findings have also been reported in other studies. For example, Wei et al demonstrated a correlation coefficient (*r* value) of 0.252 between IR and γ-GT in a Chinese population aged 20 to 74, which was slightly higher than that in our study.^[19]^ However, they utilized a homeostasis assessment model to evaluate IR. Another longitudinal study followed a relatively healthy Caucasian cohort for 3 years and further supported this relationship, with an *r* value of 0.21 for men and 0.16 for women between γ-GT and homeostasis assessment model to evaluate IR.^[20]^ The longitudinal design of this study enhanced the reliability of the conclusions. The consistently lower *r* value in our study compared to other studies can be explained by the older age and nondiabetic nature of our study cohort, as these characteristics suggest a lower IR compared to younger subjects in other studies.

Multiple factors may influence biphasic insulin secretion, such as the number of insulin granules and other metabolic signaling pathways.^[21,22]^ The role of adiposity is important to elucidate the underlying relationship between γ-GT and insulin secretion. γ-GT is recognized as a marker of intrahepatic fat accumulation.^[23]^ Furthermore, Coku et al found that γ-GT levels were related to BMI.^[24]^ Several studies have shown that individuals with higher BMI tend to have higher insulin secretion.^[25–27]^ This evidence supports our finding that γ-GT is positively correlated with both phases of insulin secretion. Interestingly, the results of the “Relationship between Insulin Sensitivity and Cardiovascular disease” study also demonstrated a similar finding.^[20]^ In their predominantly Caucasian cohort, γ-GT was associated with basal and total insulin secretion rates during the oral glucose tolerance test, with *r* values of 0.28 and 0.27 for basal insulin secretion (men and women, respectively), and 0.23 and 0.17 for total insulin secretion (men and women, respectively). Compared with their findings, the *r* values in our study were much lower. This discrepancy can be attributed to several factors. First, the participants in our study were considerably older than those in the previous study (43 and 44 years for men and women, respectively). Age-related reductions in insulin secretion may explain this difference.^[28]^ Second, the BMI of their cohort was higher than ours, and β-cell function is known to be associated with BMI.^[29]^ Hence, a higher BMI may contribute to a higher *r*-value. Lastly, the method used to quantify insulin secretion in their study was the oral glucose tolerance test, which measures a different aspect of β-cell function compared with our study. Despite these discrepancies, we can conclude that γ-GT is positively associated with insulin secretion.

It is worth noting that, although there is evidence highlighting the important role of GE in T2DM, no known study has focused on the correlation between γ-GT and GE. Our study is the first to establish a positive relationship between the 2 factors, with γ-GT exhibiting the strongest correlation compared to the other 3 factors. The mechanism underlying this interaction may involve free fatty acids (FFA). FFA has been linked to increased adiposity,^[30]^ and various hypotheses have been proposed to explain this observation, such as the “visceral fat,” “adipose tissue expandability,” or “lipid overflow” theories. Moreover, hyperglycemia suppresses lipolysis in individuals with poor glucose tolerance, leading to a decrease in plasma FFA levels. However, due to insulin resistance and the effects of glucose on the liver and muscle, FFA levels increase in these individuals.^[31]^ Hawkins et al successfully demonstrated that higher FFA levels inhibited GE.^[32]^ However, their cohort size was relatively small. Based on these pieces of evidence, it can be concluded that FFA may serve as a bridge between GE and γ-GT. Further studies with more accurate methods and larger cohorts are needed to confirm the role of γ-GT in GE.

Our study had several limitations. First, it is a cross-sectional study that provides less robust information than longitudinal studies. Second, we employed equations developed by our group, which are less accurate than gold-standard methods, such as clamp and frequently sampled intravenous glucose tolerance tests. However, obtaining all 4 parameters in the same individuals within a large cohort is challenging. A substantial number of participants in our study compensated for the accuracy of the methods.

## 5. Conclusions

In conclusion, our study demonstrated a positive relationship between γ-GT and IR, FPIS, and SPIS, and a negative association with GE in elderly Chinese individuals. Among the 4 factors, GE exhibited the highest correlation, followed by IR, SPIS, and FPIS.

## Acknowledgments

All authors would like to thank all participants of the study.

## Author contributions

**Conceptualization:** Chih-Tsueng He, Fang-Yu Chen, Chun-Heng Kuo, Chung-Yu Lin, Dee Pei, Pietro Pitrone, Jin-Shuen Chen, Chung-Ze Wu.

**Data curation:** Chih-Tsueng He, Fang-Yu Chen, Chun-Heng Kuo, Chung-Yu Lin, Chung-Ze Wu.

**Formal analysis:** Chih-Tsueng He, Fang-Yu Chen, Chun-Heng Kuo, Chung-Yu Lin, Dee Pei, Pietro Pitrone, Jin-Shuen Chen, Chung-Ze Wu.

**Investigation:** Chih-Tsueng He, Fang-Yu Chen, Chun-Heng Kuo, Chung-Yu Lin, Dee Pei, Pietro Pitrone, Jin-Shuen Chen, Chung-Ze Wu.

**Methodology:** Fang-Yu Chen, Chun-Heng Kuo, Dee Pei, Pietro Pitrone, Jin-Shuen Chen, Chung-Ze Wu.

**Project administration:** Dee Pei.

**Supervision:** Chung-Ze Wu.

**Validation:** Chih-Tsueng He, Fang-Yu Chen, Chun-Heng Kuo, Chung-Yu Lin, Dee Pei, Pietro Pitrone, Jin-Shuen Chen, Chung-Ze Wu.

**Writing – original draft:** Chih-Tsueng He, Chung-Ze Wu.

**Writing – review & editing:** Chih-Tsueng He, Chung-Ze Wu.
